# Trogocytosis-mediated immune evasion in the tumor microenvironment

**DOI:** 10.1038/s12276-024-01364-2

**Published:** 2025-01-01

**Authors:** Jeonghyun Kim, Soyeon Park, Jungseo Kim, Yewon Kim, Hong Min Yoon, Bima Rexa Rayhan, Jaekwang Jeong, Alfred L. M. Bothwell, Jae Hun Shin

**Affiliations:** 1 Institute of Advanced Bio-Industry Convergence, Yonsei University, Seoul, Korea; 2 Integrative Science and Engineering Division, Underwood International College, Yonsei University, Incheon, 21983 Korea; 3 Internal Medicine, Yale University School of Medicine, New Haven, CT 06520 USA; 4 Department of Pathology, Microbiology and Immunology, University of Nebraska Medical Center, 505 S. 45th Street, Omaha, NE 68198 USA; 5 Department of Immunobiology, Yale University School of Medicine, New Haven, CT 06520 USA

**Keywords:** Immunoediting, Immunoediting

## Abstract

Trogocytosis is a dynamic cellular process characterized by the exchange of the plasma membrane and associated cytosol during cell-to-cell interactions. Unlike phagocytosis, this transfer maintains the surface localization of transferred membrane molecules. For example, CD4 T cells engaging with antigen-presenting cells undergo trogocytosis, which facilitates the transfer of antigen-loaded major histocompatibility complex (MHC) class II molecules from antigen-presenting cells to CD4 T cells. This transfer results in the formation of antigen-loaded MHC class II molecule-dressed CD4 T cells. These “dressed” CD4 T cells subsequently participate in antigen presentation to other CD4 T cells. Additionally, trogocytosis enables the acquisition of immune-regulatory molecules, such as CTLA-4 and Tim3, in recipient cells, thereby modulating their anti-tumor immunity. Concurrently, donor cells undergo plasma membrane loss, and substantial loss can trigger trogocytosis-mediated cell death, termed trogoptosis. This review aims to explore the trogocytosis-mediated transfer of immune regulatory molecules and their implications within the tumor microenvironment to elucidate the underlying mechanisms of immune evasion in cancers.

## Trogocytosis Enhances Immune Regulation in the Tumor Microenvironment

Trogocytosis is a process where cells exchange membrane proteins, first noted in the 1970s, and it affects immune responses. Membrane proteins are molecules that help cells communicate and interact. Researchers emphasized its importance in how immune cells interact. This review discusses trogocytosis in the tumor microenvironment, influencing both immune and cancer cells. The study explains that trogocytosis allows cells to “nibble” on others, transferring membrane properties, which can change the function of immune cells like T cells and NK cells. Various methods were used to study these interactions, focusing on immune and cancer cells. Findings indicate that trogocytosis can either suppress or enhance immune responses, affecting cancer therapies such as CAR-T cells. Understanding trogocytosis could improve cancer treatments by adjusting immune responses, and future research might explore its mechanisms to boost immunotherapy. This summary was initially drafted using artificial intelligence, then revised and fact-checked by the author.

## Introduction

The concept of trogocytosis-mediated membrane protein transfer was first described in the early 1970s when B cell-derived immunoglobulins were identified on the surface of mouse thymus cells and activated T cells^1,2^. By 1999, researchers documented the transfer of peptide and MHC class I molecules from antigen-presenting cells to CD8-positive T cells^3^. In 2003, Joly and Hudrisier further demonstrated trogocytosis at the immunological synapse between B cells, T cells, and natural killer (NK) cells^4^. This phenomenon is not restricted to lymphocytes. Macrophages, dendritic cells, basophils, innate lymphoid cells, and even non-immune stromal cells can engage in trogocytosis. Additionally, cancer cells acquire immune regulatory molecules from CD4 T cells during cell-to-cell contact^5^. In this review, we discuss the process and consequences of trogocytosis, particularly in the tumor microenvironment, among immune cells and tumor cells.

## General consequences of trogocytosis

**Fig. 1:**
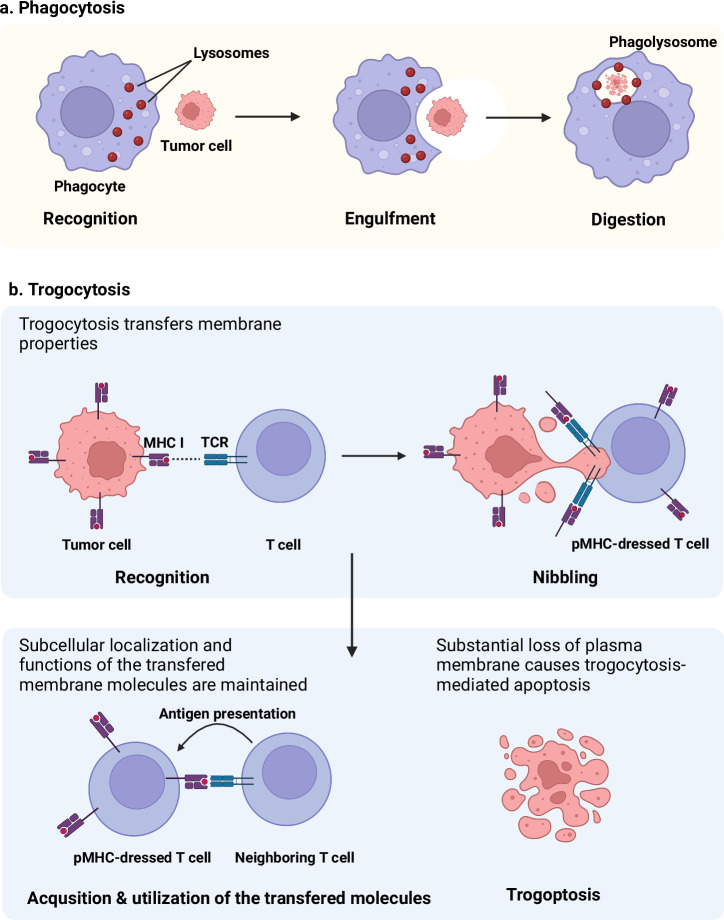
Trogocytosis and trogoptosis. **a** Phagocytes engulf their target cells, leading to the lysis of the engulfed cells via phagocytosis. **b** In contrast, trogocytosis is a distinct cellular process in which one cell “nibbles” at another during cell-to-cell contact. This nibbling affects the membrane properties. Importantly, the transferred molecules retain their membrane localization and functions. The nibbling cell acquires functional membrane molecules, whereas the nibbled cell loses portions of its membrane and attaches to the cytosol. Substantial loss of the plasma membrane can lead to apoptosis, a process referred to as trogoptosis.

Trogocytosis involves one cell “nibbling on” another to transfer membrane and cytosolic properties. This transfer allows trogocytic cells to gain functional molecules that are consequently lost by their counterparts. Significant membrane loss through trogocytosis can lead to cell rupture and death. For example, the ameba *Entamoeba histolytica* uses trogocytosis to kill host intestinal epithelial cells, which aids infection^6^. This process is driven by interactions between amebic lectins and host receptors, which leads to cytoskeletal remodeling. Amoebae use trogocytosis to kill living cells, whereas dead cells are engulfed by phagocytosis. Moreover, neutrophils target live *T. vaginalis* parasites via trogocytosis^7^. Macrophages and neutrophils eliminate antibody-coated cancer cells through trogoptosis, a form of trogocytosis-mediated apoptosis^8,9^. Neutrophils also use trogocytosis to reduce sperm dissemination^10^. Significant membrane loss can result in trogoptosis, which is also known as trogocytosis-mediated apoptosis^11^. Partial membrane loss through trogocytosis significantly alters cellular functions by reducing functional membrane proteins. Trogocytic cells gain these functions, unlike phagocytosis, which degrades transferred molecules (Fig. 1). Trogocytosis preserves protein functionality, which poses challenges for cell identification via surface markers^12^. For example, T cells can acquire CD20, a B cell-specific marker, which complicates conventional cell identification methods^13^. Recent studies have shown that trogocytosis affects immune cell functions by redistributing functional membrane proteins^14^. This redistribution impacts antigen presentation, immune activation, and cell signaling^15,16^. The gain or loss of membrane properties through trogocytosis leads to significant changes in cellular functions.

## T cell trogocytosis in the tumor microenvironment

**Fig. 2:**
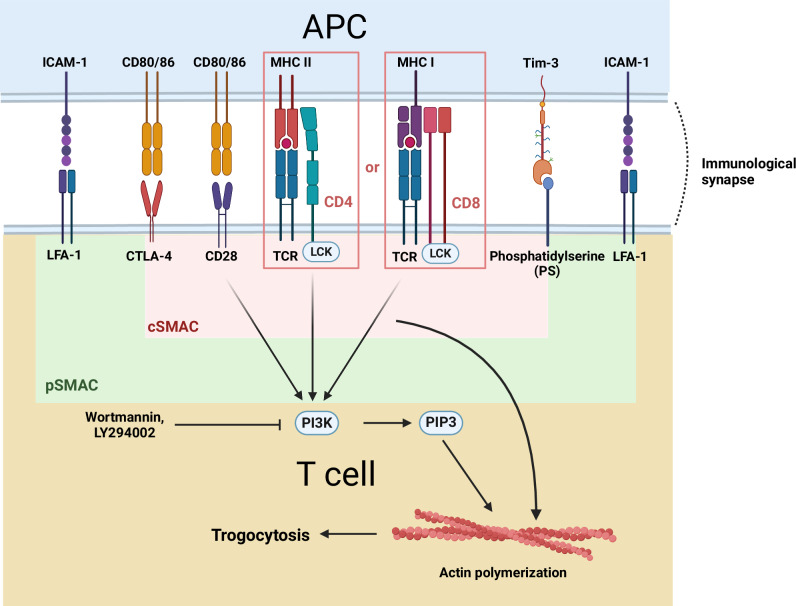
Underlying mechanisms of trogocytosis in T cells. Trogocytosis is initiated by ligand‒receptor binding, which involves adhesion molecules, such as ICAM-1 and LFA-1, and other cell-specific molecules. T-cell trogocytosis occurs in a TCR affinity-dependent manner. CD28 binding to CD80/86 also triggers trogocytosis. TCR and CD28 stimulation activates the PI3K pathway, leading to cytoskeleton remodeling via actin polymerization, a key mechanism in trogocytosis. PI3K inhibitors, such as wortmannin and LY294002, reduce trogocytosis. Phosphatidylserine on activated T cells binds to Tim-3 on dendritic cells (DCs) to aid the transfer of peptide-loaded MHC molecules to T cells. Regulatory T cells capture CD80/86 on APCs through CTLA-4-mediated trogocytosis by depleting co-stimulatory molecules. CTLA-4 also removes endogenous and trogocytosed CD80/86 on T cells via cis-endocytosis.

T cells play crucial roles in anti-tumor immunity in both cellular and humoral immune responses. Therefore, molecular changes in T cells or their target cells after trogocytosis significantly alter their cellular functions. Trogocytosis is initiated by the engagement of ligands and receptors in two cells in contact. Interactions between adhesion molecules and integrins at a peripheral supramolecular activation cluster (pSMAC) form an immunological synapse between T cells and antigen-presenting cells (APCs), followed by the engagement of the T cell receptor (TCR) with peptide-loaded major histocompatibility complex (pMHC) molecules at a central supramolecular activation cluster (cSMAC)^17^. This process not only activates TCR signaling but also induces T cell trogocytosis, which involves the transfer of membrane properties, including pMHC, from APCs to T cells^18^. T cells perform trogocytosis in an antigen-specific and TCR affinity-dependent manner^19,20^. TCR stimulation activates Lck and Syk kinases, as well as phosphatidylinositol-3-kinase (PI3K), leading to actin polymerization and subsequent trogocytosis^21^ (Fig. 2). Inhibition of the PI3K pathway or actin polymerization by wortmannin or LY294002, respectively, reduces trogocytosis in mouse T cells^22^. Additionally, the binding of co-stimulatory or inhibitory molecules, such as B7 molecules and phosphatidylserine (PS), to their receptors CD28 and Tim-3 promotes T cell trogocytosis, whereas CTLA4 counteracts this effect via cis-endocytosis of surface molecules^19,23–25^. However, the types of T cells that perform trogocytosis and the underlying mechanisms of trogocytosis are still under investigation.

### Fratricide of tumor-infiltrating cytotoxic T lymphocytes

CD8 T cells perform trogocytosis to acquire pMHCs from antigen-presenting cells or tumor cells in contact with each other in a TCR-dependent manner^3^. This process results in the generation of pMHC-dressed CD8 T cells that can present the transferred antigens to other neighboring activated CD8 T cells^26,27^. *Dionne* et al. reported that human CD8 T cell lines were able to present a human melanoma-derived antigen, the gp100-derived epitope g209-2 M, to other CD8 T cells^28^. Antigen presentation by CD8 T cells leads to IFN-γ secretion and the proliferation of other CD8 T cells, followed by the lysis of pMHC-dressed CD8 T cells. This antigen-specific killing of pMHC-dressed CD8 T cells by neighboring CD8 T cells is known as fratricide^3,28^.

Co-culture of melanoma-derived pMHC-dressed CD8 T cells with their fraternal CD8 T cells, which have the same TCR or a different TCR recognizing the same antigen, elevated the levels of cleaved caspase-3, a marker of apoptosis, in pMHC-dressed CD8 T cells^29^. A recent study also revealed that tumor antigen-specific CD8 tumor-infiltrating lymphocytes (TILs) can acquire pMHC molecules from APCs in a Tim-3/phosphatidylserine (PS)-dependent fashion^25^. PS on the surface of CD8 T cells interacts with Tim-3 on dendritic cells to trigger the trogocytic transfer of pMHCs. Following the acquisition of tumor pMHCs from APCs, pMHC-dressed CD8 T cells undergo fratricide by neighboring CD8 T cells. Using an anti-Tim-3 monoclonal antibody to block Tim-3 from binding to PS reduced trogocytosis, which increased the efficacy of T cell-mediated tumor cell killing^25^. These findings suggest that the trogocytosis-mediated fratricide of cytotoxic T cells impedes their anti-tumor immunity to generate an immunosuppressive tumor microenvironment. Notably, recent studies have reported that trogocytosis-mediated fratricide also reduces the therapeutic efficacy of chimeric antigen receptor T (CAR-T) cell therapy^30^. This phenomenon will be further discussed below in the section on CAR-T cell trogocytosis.

### Enhanced anti-tumor reactivity of tumor-infiltrating lymphocytes after trogocytosis

Trogocytosis between T cells and tumor cells has been reported in melanoma and myeloma^28,31^. Peripheral blood mononuclear cell (PBMC)-derived CD8 T cells acquire membranes from autologous melanoma cells or cell lines in contact with them^32^. This trogocytic membrane transfer is T cell receptor-dependent, epitope-specific, and associated with highly cytotoxic clonal subsets. Adoptive transfer of these membrane-captured CD8 T cells into human melanoma-bearing mice inhibited tumor progression. *Eisenberg* et al. further confirmed that both CD4 and CD8 T cells in the PBMCs of melanoma patients and tumor-infiltrating lymphocytes (TILs) that capture melanoma antigens show preferential reactivity and cytotoxicity against melanoma^33^. CD4 and CD8 T cells also acquire H-Ras oncoproteins from melanoma cells via trogocytosis in a TCR-dependent manner^34^. Co-culture of a human melanoma cell line, MEL526 cells expressing GFP-tagged H-Ras, with TILs or CD3 T cells from PBMCSs resulted in H-ras transfer from melanoma cells to T cells, which augmented T cell activation, including IFN-γ secretion, and T cell cytotoxicity.

Furthermore, CD4 T cells can acquire NK cell-activating ligands, such as NKG2DL and NKp46L, from human melanomas, allowing them to activate NK cells in the tumor microenvironment^35^. Although trogocytosis enables antigen presentation and cell stimulation in vitro, it can result in trogocytosis-mediated cell death, such as fratricide, or dysregulated target cell activation in vivo. In other words, the environmental context of the involved cell types and molecules on the cell surface is definitive for the outcomes of trogocytosis.

### Transfer of immune regulatory molecules via trogocytosis

Trogocytosis not only transfers pMHCs but also various immune regulatory molecules during cell-to-cell contact. The acquisition of the co-stimulatory ligands CD80 (B7.1) and OX40L on the surface of CD4 T cells by membrane transfer was reported in 2001^23,36^. These findings were confirmed in CD28 knockout mice and FLAG-tagged-OX40L-expressing COS-1 cells. In 2007, LeMaoult et al. reported that CD8 T cells acquired human leukocyte antigen G (HLA-G) from encountering APCs^37^. HLA-G is a non-classical MHC class I molecule with immune-suppressive functions^38^. Therefore, the acquisition of HLA-G by CD8 T cells alters their functions such that they resemble regulatory T cells (Tregs)^37^. Elevated expression of HLA-G has been reported in various cancers, including melanoma and myeloma, and is associated with poor prognosis. Interestingly, trogocytosis is more common in multiple myeloma than in chronic lymphocytic leukemia and Waldenstrom macroglobulinemia, and T cells are preferred recipients of trogocytosis over B cells and NK cells^39^. In human multiple myeloma, the regulatory potency of HLA-G-acquired effector T cells is similar to that of natural regulatory T cells (nTregs). In addition, *Gary* et al. reported that human CD8 T cells acquire PD-L1 from encountered dendritic cells and melanoma cells in an antigen-specific manner^40^. As a result, PD-L1-acquired trogocytic CD8 T cells induced the apoptosis of neighboring CD8 T cells that express the PD-L1 receptor PD1. Overall, the transfer of immune stimulatory or regulatory molecules—CD80, CD86, OX40L, HLA-G, and PD-L1—via trogocytosis significantly inhibits anti-tumor immunity and contributes to the development of the immune-suppressive tumor microenvironment.

Like other T cells, regulatory T cells (Tregs) also perform trogocytosis^41^. Antigen-specific regulatory T cells (Tregs) activated by antigen-pulsed dendritic cells (DCs) suppress naïve T cells. Strong binding of Tregs to pMHC class II molecules expressed on DCs resulted in the depletion of pMHC class II molecules on the surface of DCs, which reduced their antigen presentation potential^42^. Induced regulatory T cells (iTregs) generated from CD80^-/-^CD86^-/-^ double knockout mice undergo trogocytosis and acquire CD80 and CD86 from engaged DCs^43^. This trogocytosis occurred in a CTLA4-, CD28- and PDL1-independent manner. Notably, compared with iTregs that did not acquire CD86, CD80/86-dressed iTregs more strongly suppressed the proliferation of naïve CD4 T cells. Additionally, Tregs express CD137, a receptor for the co-stimulatory ligand CD137L. During the cell-to-cell interaction between APCs and Tregs, CD137L binding to CD137 undergoes trogocytic transfer of CD137L to Tregs, followed by CD137L depletion in APCs^44^. Furthermore, Treg trogocytosis depletes CD80/86 in APCs in a CTLA4-dependent manner^24^. The binding of CTLA4 to its ligand CD80/86 on the surface of mouse B cells and dendritic cells triggers the transfer of CD80/86 to mouse Tregs. This transfer interrupts co-stimulation and disrupts the cis-CD80/PD-L1 heterodimer formation, which increases free PD-L1 on APCs^24^. These findings suggest the tumor-promoting potential of Treg trogocytosis, which depletes pMHC and co-stimulatory molecules on APCs, interrupting T cell activation in the tumor microenvironment.

### Altered T cell differentiation after trogocytosis

Overall, CD4 T cell-mediated trogocytosis contributes to immunosuppression in the tumor microenvironment. As mentioned above, trogocytosis generates pMHC class II-dressed CD4 T cells together with CD80/86 transfer^31^. pMHC class II molecules and CD80/86-dressed CD4 T cells can present antigens and provide co-stimulation using the transferred molecules to other CD4 T cells^45^. To test the potential of trogocytosis among different CD4 T cell subtypes, *Reed* et al. performed CD4 T cell co-culture using peptide-pulsed bone marrow-derived dendritic cells and transfected mouse fibroblasts expressing antigenic pMHC molecules^46^. Notably, trogocytosis-positive CD4 T cells expressed T helper type 2 (Th2) cytokines along with GATA3, a Th2-specific transcription factor. CD4 T cells that experienced trogocytosis during in vitro co-culture with APCs presented a decrease in the expression of IFN-γ from 13.4% to 1.5%, whereas the expression of the Th2 cytokine IL-4 shifted to 77.4%. Moreover, Th2-polarized CD4 T cells presented increased trogocytosis compared with T helper type 1 (Th1) or non-polarized CD4 T cells. This finding has been confirmed in vivo, revealing a Th2 phenotype of trogocytosed CD4 T cells in wild-type and TCR-transgenic mice. Furthermore, basophils also undergo trogocytosis to acquire pMHC class II from APCs^47^. pMHC-dressed basophils present antigens and provide IL-4 to naïve CD4 T cells, which promotes Th2 differentiation. Because Th2 polarization inhibits Th1 differentiation and IFN-γ secretion, the trogocytosis-mediated Th2 polarization of CD4 T cells inhibits Th1-mediated anti-tumor immunity, which contributes to the development of the immunosuppressive tumor microenvironment.

**Fig. 3:**
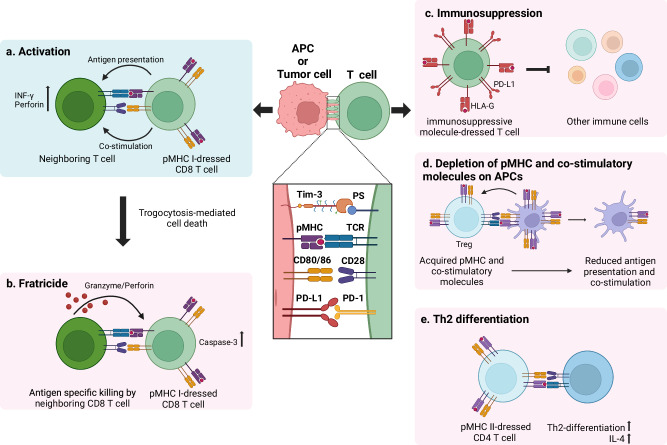
Consequences of T cell trogocytosis. The acquisition of immune regulatory molecules by T-cell trogocytosis modulates anti-tumor immunity. **a** *Activation*: T cells acquire peptide‒MHC (pMHC) complexes from antigen-presenting cells (APCs) or tumor cells, enabling them to function as APCs. This process leads to the activation of neighboring T cells. **b** *Fratricide*: CD8 T cells that acquire pMHC I complexes can become targets of antigen-specific killing by neighboring CD8 T cells, leading to fratricide. **c** *Immunosuppression*: The immune regulatory molecules transferred after trogocytosis, such as HLA-G and PD-L1, suppress the reactivity of other immune cells. **d** *Depletion of pMHC and co-stimulatory molecules on APCs*: Regulatory T cells (Tregs) can capture co-stimulatory molecules from APCs via CTLA-4-mediated trogocytosis, leading to the depletion of these molecules on APCs. **e** *Th2 differentiation*: Trogocytosis of CD4 T cells dressed with pMHC complexes can induce the differentiation of neighboring T cells into Th2 cells, altering the immune response toward a Th2 phenotype.

Collectively, tumor-infiltrating lymphocytes (TILs) acquire pMHCs and various co-stimulatory or inhibitory molecules from cells they encounter, including tumor cells (Fig. 3). Although trogocytosis allows T cells to become antigen-presenting cells, antigen presentation by pMHC-dressed T cells can inhibit the anti-tumor immune response. (1) Trogocytosis-mediated generation of pMHC-dressed CD8 T cells causes their fratricide by neighboring CD8 T cells. (2) Antigen presentation by pMHC-dressed CD4 T cells is not compatible with antigen presentation by APCs, resulting in Th2-skewed differentiation. (3) T cells acquire various immunoregulatory molecules, such as HLA-G, via trogocytosis. (4) Co-stimulatory molecules and pMHC molecules on APCs are depleted by trogocytosis.

## NK cell trogocytosis in the tumor microenvironment

Natural killer (NK) cells, which are cytotoxic innate lymphocytes, also undergo functional changes via trogocytosis in the tumor microenvironment^16^. NK cells interact with their target cells by forming an immunological synapse, where activation or inhibitory receptors bind to their ligands^48^. For example, killer Ig-like receptors at the surface of NK cells play inhibitory roles by recognizing MHC class I molecules and clustering them at the immunological synapse. This clustering serves as a platform for intracellular signal transduction to inhibit NK cell cytotoxicity. Natural Killer Group 2 membrane D (NKG2D) is an activation receptor expressed on NK cells, and its ligand, MHC I-related chain A (MICA), is highly expressed on various tumors, including bone marrow myeloma cells^49^. The interaction between NKG2D and MICA activates NK cells, followed by the subsequent lysis of target cells that express MICA. Engagement of NKG2D induces NK cells to secrete cytotoxic granules, such as perforin and granzyme, into the target cells^50^. Importantly, this ligand‒receptor binding at the immunological synapse triggers trogocytosis, which allows NK cells to acquire MICA from their targets. MICA-acquired NK cells can interact with NKG2D on neighboring NK cells to trigger NK cell fratricide. Similarly, NK cell fratricide occurs when NK cells acquire retinoic acid early-inducible protein 1 (Rae-1), another tumor-derived ligand for NKG2D, through trogocytosis^51^. These findings imply the negative effects of trogocytosis on anti-tumor immunity.

Similar to T cells, NK cells can acquire the immune regulatory protein HLA-G from the HLA-G1-transfected melanoma cell line M8-HLA-G1 via trogocytosis^52^. HLA-G1-dressed NK cells interact with neighboring NK cells via the cognate receptor ILT2, which reduces NK cell proliferation and cytotoxicity. The transferred HLA-G1 on NK cells binds to ILT2 on other NK cells to transmit inhibitory signals that suppress NK cell proliferation and cytotoxicity. *Gonzalez* et al. reported decreased cytokine production in NK cells due to the trogocytic transfer of CD9 from co-cultured tubo-ovarian high-grade serous carcinoma (HGSC) cells^53^. Although the role of CD9 in immunity remains unclear, CD9 CRISPR knockout and treatment with an anti-CD9 blocking antibody restored NK cell cytotoxicity in vitro, suggesting the immune-suppressive function of CD9 in NK cells following trogocytosis. Similarly, NK cells can acquire PD-1 from C1498 leukemia cells via trogocytosis, which is mediated by SLAM receptors and inhibits their anti-tumor activity^54^. This inhibition could be rescued by PD-1 blockade, thereby enhancing NK cell cytotoxicity against tumors and providing insight into why checkpoint blockade therapy relies on NK cells despite their typically low expression of PD-1.

Trogocytosis of NK cells at immunological synapses can be inhibited by blocking the Src kinase pathway using the inhibitor PP2^55^. The Src kinase pathway is crucial for the recruitment of NK cell receptors and their ligands to immunological synapses, thereby facilitating trogocytosis. In addition, NK cell trogocytosis involves actin cytoskeleton remodeling and depends on ATP, Ca^2+^, and PKC^56^. NK cells not only interact with tumor cells but also gain or lose surface molecules during interactions with other cells, including APCs. For example, recent studies have shown that NK cells acquire MHC class II and the co-stimulatory molecules CD80/86 from DCs via trogocytosis^55^. These pMHC-dressed NK cells can present antigens to CD4 T cells. However, this antigen presentation does not reach functional levels and thereby hinders T-cell immune responses. Furthermore, NK cell trogocytosis can capture the CCR7 chemokine receptor from allogeneic DCs and T cells to facilitate NK cell migration into secondary lymphoid organs^57^.

**Fig. 4:**
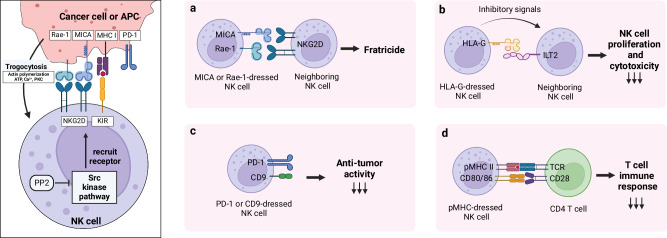
Trogocytosis by NK cells. Natural killer (NK) cells undergo trogocytosis via interactions between NK cell receptors, such as NKG2D and KIR, and their corresponding ligands. The recruitment of these receptors to the membrane is regulated by the Src kinase pathway; thus, inhibiting Src with PP2 reduces trogocytosis. After trogocytosis, NK cells acquire ligands, such as MICA, Rae-1, or peptide-loaded MHC class I molecules, from tumor cells or antigen-presenting cells (APCs). **a** MICA- or Rae-1-dressed NK cells are targeted by neighboring NK cells, leading to fratricide. **b** NK cells acquire immune regulatory molecules such as HLA-G from tumor cells, inhibiting the proliferation and cytotoxicity of other NK cells. **c** The acquisition of CD9 or PD-1 directly downregulates NK cell reactivity. **d** NK cells acquire pMHC II and CD80/86 from dendritic cells, presenting antigens to CD4 T cells, but this antigen presentation is less effective than that of professional APCs, leading to reduced T cell responses.

In summary, NK cell trogocytosis plays a crucial role in modulating immune responses within the tumor microenvironment (Fig. 4). Through the acquisition of immune regulatory proteins such as HLA-G, CD9, and PD-1, NK cells can undergo significant changes in their cytotoxicity and proliferation. The Src kinase pathway, along with factors such as ATP, Ca^2+^, and PKC, is involved in trogocytosis at immunological synapses. Moreover, the acquisition of molecules such as MHC class II and CD80/86 by NK cells further influences T-cell responses. Understanding the complex dynamics of NK cell trogocytosis and its impact on immune regulation provides valuable insights into anti-tumor immunity.

## Trogocytosis of other immune cells

**Fig. 5:**
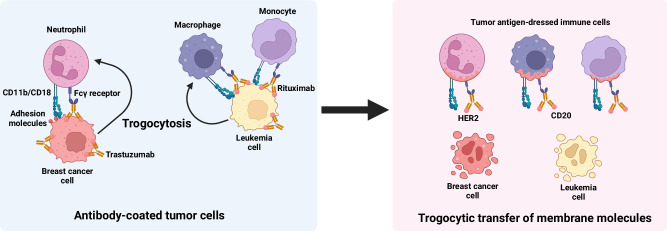
Trogocytosis of antibody-coated tumor cells by macrophages, monocytes, and neutrophils. Neutrophils, macrophages, and monocytes undergo trogocytosis when in contact with antibody-coated tumor cells. Interactions between CD11b/CD18 integrins and adhesion molecules on the tumor cell surface establish a cytotoxic synapse. At this synapse, the Fc gamma receptor (FcγR) binds to trastuzumab bound to HER2, initiating trogocytosis and transferring membrane molecules from breast cancer cells to innate immune cells. A similar process occurs with rituximab-coated leukemia cells. Notably, significant tumor cell membrane loss leads to trogocytosis-mediated apoptosis, known as trogoptosis.

Neutrophils play a pivotal role in the elimination of antibody-coated cancer cells via a process termed trogoptosis^9,58^ (Fig. 5). They nibble and ingest fragments of the target cell membrane, which leads to trogocytosis-mediated apoptosis of the target cells. This mechanism hinges on the engagement of Fcγ receptors (FcγRs) with antibody-bound tumor cells and the adhesive interaction mediated by CD11b/CD18 integrins. Blockades of Fcγ receptors, particularly FcγRIIa and CD11b/CD18 integrins compromise antibody-dependent cellular cytotoxicity^9^. Conversely, inhibition of the ‘Don’t eat me’ signal CD47 potentiates active mechanical disruption of cancer cell membranes and necrotic target cell death^59,60^. Bouti et al. reported that the inhibition of CD47 signaling activated CD11b/CD18 integrins to form cytotoxic synapses between neutrophils and tumor cells and lead to mechanical membrane disruption and trogoptosis^60^.

Similarly, monocytes and macrophages also perform trogocytosis in antibody-coated tumor cells^61,62^ (Fig. 5). Rituximab treatment of lymphocytic leukemia induced substantial loss of CD20 on B cells via trogocytosis by monocytes^63^. The binding of trastuzumab to human epidermal growth factor receptor 2 (HER2) leads to the death of antibody-opsonized breast cancer cells via monocyte- and macrophage-mediated trogocytosis^8,11^. Recent advances have suggested that the ability of macrophages to undergo trogocytosis depends on the density of FcγR and incomplete encircling of the target cell^64,65^. Consistent with findings in neutrophils, blockade of CD47 in renal cell carcinoma enhanced macrophage trogocytosis^66^. Overall, trogocytosis of antibody-opsonized tumor cells via FcγR interactions enhanced the tumoricidal activity of neutrophils, monocytes, and macrophages.

Recently, Kim et al. reported that trogocytosis transfers membrane particles from T-cell microvilli onto the surface of cognate antigen-bearing APCs^67^. This process delivers T-cell receptors (TCRs) at all stages of T-cell activation to APCs, which leads to the loss of membrane TCRs and microvilli-associated proteins and lipids in T cells. Interestingly, this phenomenon results in the rapid recovery of surface TCRs in T cells and further promotes the clonal expansion of T cells via the reprogramming of lipid metabolism^68^.

## Tumor Cell-mediated Trogocytosis

Tumor cells also employ trogocytosis as a strategy to modulate immune responses, as demonstrated in recent studies. *Shin* et al. reported that both mouse and human colon cancer cells can acquire immune cell-specific markers, such as CD4 and CD45, from CD4 T cells within the tumor microenvironment^5^. This process was inhibited by treatment with the PI3 kinase and actin polymerization inhibitors wortmannin and latrunculin A, respectively, which suggested that the acquisition of CD4 and CD45 by cancer cells occurs via trogocytosis. Notably, this phenomenon was observed in malignant colon cancer cells but not in normal epithelial cells. In a murine model of liver metastasis established via a splenic injection of colon cancer organoids into C57BL/6 mice, cancer cells were found to acquire various immune regulatory molecules, including CTLA-4, PD-L1, PD-1, Tim-3, VISTA, LAG-3, and CD38, through trogocytosis. This transfer of immune regulatory molecules significantly enhanced the immunosuppressive functions of colon cancer cells. When these trogocytic cancer cells were co-cultured with syngeneic mouse splenocytes, Th1-mediated antitumor immunity markedly decreased compared with that in co-cultures involving non-trogocytic cancer cells. These observations suggest that tumor trogocytosis contributes to the formation of the immunosuppressive tumor microenvironment.

*Shin* et al. further investigated the impact of trogocytosis on tumor antigen presentation in colon cancer. In their murine liver metastasis model, cancer cells acquired B7.1 and B7.2 molecules from the tumor microenvironment, along with MHC class II molecules. Interestingly, although the levels of B7.1 and B7.2 in trogocytic cancer cells were comparable to those in CD11b-expressing immune cells within the tumor microenvironment, the transfer of MHC class II molecules was less efficient. This inefficiency could suggest a reduction in antigen presentation capacity due to the loss of B7.1 and B7.2 from antigen-presenting cells, which may impair the activation of anti-tumor CD4 T cells and contribute to the development of an immunosuppressive tumor microenvironment.

More recently, trogocytosis by tumor cells has been implicated in the capture of chimeric antigen receptor (CAR) molecules from CAR-T cells, as reported in studies using human glioblastoma and prostate adenocarcinoma cell lines^69^. This phenomenon results in CAR-T-cell dysfunction and antigen escape. These findings highlight the role of trogocytosis in facilitating immune evasion and promoting tumor progression, underscoring its potential as a target for therapeutic intervention.

## Trogocytosis in CAR-T and CAR-NK cell therapy

Chimeric antigen receptors (CARs) are genetically engineered antigen receptors that modify the specificity and function of T cells and other immune cells toward a selected target, allowing interactions without the need for MHC-mediated antigen presentation^70,71^. CAR-T and CAR-NK cell therapies have shown promising clinical outcomes in the treatment of cancer, as exemplified by the FDA approval of CAR-T cell therapy for B cell malignancy^72,73^. Despite these breakthroughs, challenges such as metabolic disruption, cellular exhaustion, and trogocytosis remain, which limit their effectiveness in the tumor microenvironment^74–78^.

### Trogocytosis-mediated loss of tumor antigens

**Fig. 6:**
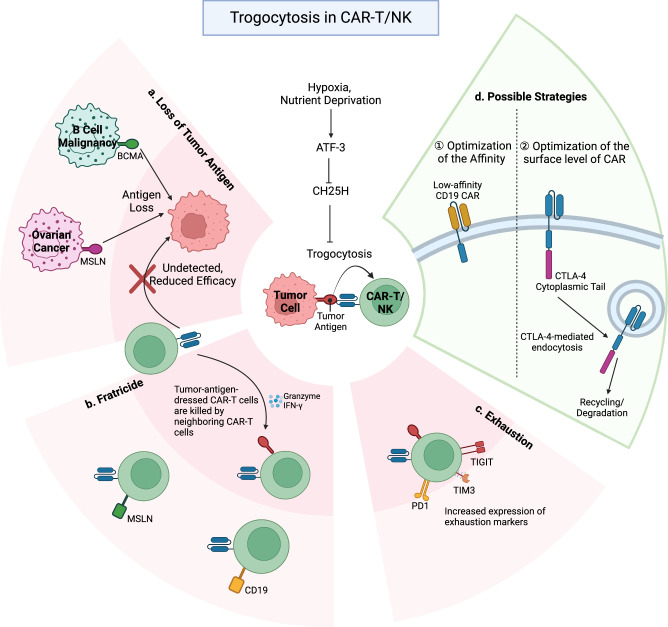
Adverse effects of trogocytosis in CAR-T cell therapy. CAR-T cell trogocytosis disrupts CAR-T cell therapy in the following ways. **a** CAR-T cells acquire CAR-targeted antigens, such as CD19, BCMA, and MSLN, from leukemia, myeloma, and ovarian cancer cells, respectively, to generate target-free tumor cells. **b** Tumor antigen-dressed CAR-T cells are attacked by other CAR-T cells, leading to fratricidal cell death. **c** Prolonged exposure to tumor antigens causes CAR-T cell exhaustion. Trogocytic CAR-T cells, due to their strong antigen affinity, exhibit increased expression of exhaustion markers, such as Tim-3, TIGIT, and PD1. **d** To counter these adverse effects, low-affinity CD19 CAR-T cells were developed to minimize trogocytosis in the context of B-cell lymphoma. Additionally, fusing the cytoplasmic tail of CTLA-4 to the CAR reduces surface CAR levels, optimizing CAR-T cell therapeutic efficacy.

Recent studies have demonstrated that trogocytosis occurs between cancer cells and CAR-T cells to reduce antigen density on target cancer cells and decrease the efficacy of CAR-T-cell therapy^30,78–80^ (Fig. 6). For example, B cell maturation antigen (BCMA) is a widely used target antigen for CAR-T-cell therapy in B cell malignancies^81^. Raje et al. demonstrated that anti-BCMA CAR-T-cell therapy has a limited duration and often leads to relapse in multiple myeloma patients^82^. Similarly, Camviel et al. further reported that CAR-T cells trogocytose and internalize BCMA from myeloma cells, which leads to BCMA loss in myeloma cells and reduces the therapeutic efficacy of CAR-T cells^78^. In solid tumors, Schoutrop et al. reported that CAR-T cells that target mesothelin (MSLN) also depleted MSLN in human ovarian cancer cells via trogocytosis, which led to target antigen loss^80^.

Interestingly, recent findings have shown that the loss of target antigens in cancer cells following trogocytosis is reversible. In a NALM6 acute lymphoblastic leukemia model, Hamieh et al. showed that the decrease in CD19 expression on the surface of NALM6 cells co-cultured with anti-CD19 CAR-T cells was partially reversed after six days. Despite this recovery, the overall CD19 density remained lower in co-cultured NALM6 cells than in the single-culture control, which decreased CAR-T-cell efficacy^30^. Trogocytic transfer of CD19 was also observed in CAR-NK cells. Upon co-culture with CD19-expressing Raji lymphoma cells, Li et al. reported that CAR-NK cells rapidly acquired CD19 and other B cell-specific markers, such as CD20 and CD22^79^. Overall, these studies suggest that the therapeutic efficacy of CAR-expressing cells is closely associated with the antigen density of target cells. Despite the observed reversibility, the rapid process of trogocytosis between cancer cells and CAR-expressing cells ultimately leads to the loss of target antigens and diminished anti-tumor immune responses, which results in the survival of cancer cells and resistance to CAR-based therapies.

### Fratricide of CAR-expressing Cells

Trogocytosis-mediated membrane transfer generates pMHCs, self-antigen-dressing CAR-T cells or CAR-NK cells. These dressed CAR-T or CAR-NK cells are recognized by neighboring T cells or NK cells, which leads to fratricide^30,79,80^ (Fig. 6). In vitro co-culture of anti-CD19 CAR-T cells that had acquired CD19 from NALM6 cells through trogocytosis with fresh anti-CD19 CAR-T cells resulted in the production of IFN-γ and granzyme B, key cytokines involved in T-cell-mediated apoptosis, which indicated fratricidal attack^30^. Additionally, anti-mesothelin CAR-T cells that had acquired MSLN antigen were also susceptible to cytolysis by other neighboring MSLN-specific CAR-T cells. These fratricidal attacks may explain the decreased proliferation and viability of trogocytosed CAR-T cells compared with their counterparts^80^. A similar case of fratricidal attack was also observed in a study of CD19-specific CAR-NK cell therapy against Raji CD19 lymphoma cells. Co-culture of CD19-dressed CAR-NK cells with autologous CD19-specific CAR-NK cells led to the apoptosis of CD19-dressed CAR-NK cells^79^. Overall, the shift in the reactivity of CAR-expressing cells from targeting tumor cells to attacking other CAR-expressing cells, which is induced by trogocytosis, leads to fratricide and significantly diminishes their anti-tumor activity.

### Exhaustion of CAR-T cells via trogocytosis

In addition to hindering the viability and activity of CAR-expressing cells via fratricide, chronic antigenic stimulation by cancer cells can lead to exhaustion of effector CAR-expressing cells^83–85^ (Fig. 6). For example, CAR-NK cells repeatedly challenged by self-engagement with CD19-specific NK cells exhibit exhaustion markers, such as PD1, TIM3, and TIGIT^79^. Furthermore, compared with control T cells, MSLN-specific CAR-T cells also presented increased exhaustion marker levels^80^. These findings suggest that trogocytic CAR-expressing cells experience functional exhaustion compared with their non-trogocytic counterparts, which explains their overall reduced viability and activity against cancer cells.

Interestingly, CAR-T cells that lack cholesterol 25-hydroxylase (CH25H) expression exhibit increased trogocytosis^86^. CH25H catalyzes the monooxygenation of cholesterol into 25-hydroxycholesterol, which influences the fluidity of lipid membranes and prevents trogocytosis^86,87^. The reduction in CH25H expression is regulated by activating transcription factor-3 (ATF3), which is activated by numerous factors in the tumor microenvironment, such as hypoxia and nutrient deprivation^88^. ATF3-null CAR-T cells exhibit a reduction in exhaustion and apoptosis markers and improved anti-tumor reactivity due to increased expression of CH25H^86^. These findings highlight the importance of the ATF3-CH25H pathway in regulating the exhaustion, proliferation, and overall anti-tumor reactivity of CAR-T cells.

### Strategies to overcome CAR-T cell trogocytosis

Like T-cell trogocytosis, which occurs in a TCR affinity-dependent manner, the affinity of CAR-T cells also determines their trogocytosis^89^. Enhanced CAR-T-cell efficacy and longevity have been reported when low-affinity CD19 CAR-T cells were applied to acute lymphoblastic leukemia^89^ (Fig. 6). In 2022, Olson et al. demonstrated that low-affinity CD19 CAR-T cells presented reduced trogocytosis and antigen loss in target tumor cells both in vitro and in vivo^90^. These findings indicate a significant correlation between the affinity of CAR-T cells and trogocytosis-mediated immunosuppression. Notably, when the endocytic feature of the cytoplasmic domain of CTLA-4 is used, fusing this domain with CD19 CARs decreases the surface expression levels of CARs, which reduces trogocytosis and improves the survival of CAR-T cells^91^ (Fig. 6). Moreover, CTLA-4 fusion CAR-expressing T cells retain a stronger central memory phenotype and better persistence^91^. These findings highlight the need for a better understanding of CAR-T-cell trogocytosis to develop more effective strategies for CAR-T-cell therapies against various cancers.

## Utilization of trogocytosis

**Fig. 7:**
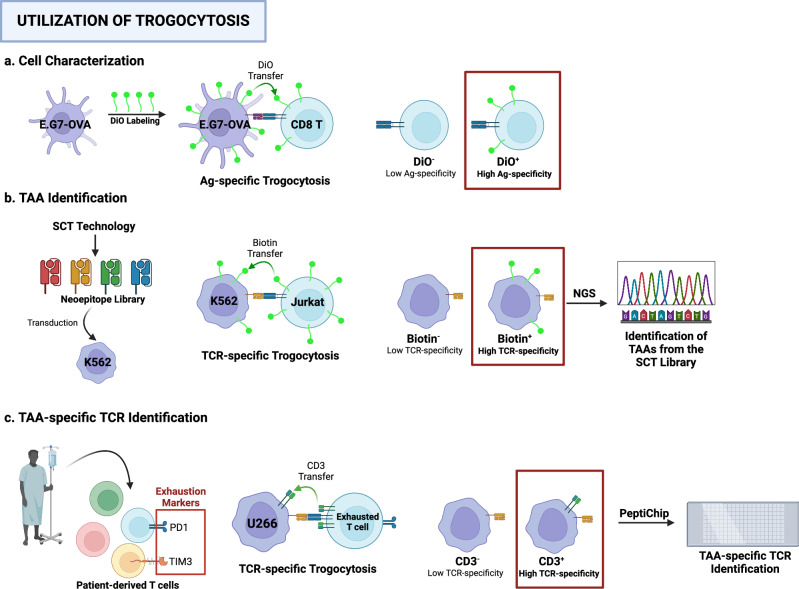
Utilization of trogocytosis. **a** Mouse thymoma EL4 cells were labeled with membrane markers, such as 3,3′-dioctadecyl-oxacarbocyanine perchlorate (DiO), and co-cultured with CD8 T cells. As a result, DiO was transferred to CD8 T cells in an antigen-specific manner through trogocytosis. The isolation of DiO+ cells enabled the characterization of antigen-specific CD8 T cells. **b** Using single-chain trimer technology, a library of tumor-associated antigens (TAAs) was generated and transduced into K562 leukemia cells. Biotin-labeled Jurkat cells were then co-cultured with K562 cells expressing TAAs. Trogocytosis involves the transfer of biotin to target K562 cells. Isolation of biotin+ cells enriched with K562 cells expressing TAAs with high TCR specificity. The selected TAAs were identified via next-generation sequencing (NGS). **c** Patient-derived T cells expressing exhaustion markers, such as PD-1 or TIM-3, showed enhanced cytotoxicity against target cells, indicating high antigen specificity. After these PD-1+ TIM-3+ T cells were co-cultured with U266 cells, trogocytosis transferred CD3 from the T cells to the U266 cells. Isolation of CD3+ U266 cells after co-culture, followed by TCR identification, can reveal TAA-specific TCRs via the PeptiChip technique.

T-cell-mediated trogocytosis is determined by the antigen specificity and affinity of T cells^16^. Higher antigen specificity increases trogocytosis-mediated membrane transfer. Recent studies focused on this aspect of T-cell-mediated trogocytosis have developed novel techniques for cellular and molecular identification (Fig. 7).

In 2006, *Puaux* et al. proposed a trogocytosis-based assay for detecting antigen-reactive CD8 T cells using flow cytometry^92^. The APCs were labeled with membrane trackers, such as DiO, and pulsed with ovalbumin (OVA_257–264_) peptide. Co-culture of the labeled APCs with mixed populations of T cells, including OVA_257–264_-specific OT-I T cells, resulted in the selective transfer of the membrane trackers from the APCs to the OT-I T cells. This assay also detects antigen-specific CD8 T cells in mice after vaccination. Furthermore, this concept is applicable to antigen-specific CD4 and B lymphocytes. Subsequent research demonstrated the purification and quantification of antigen-specific lymphocytes, as well as their characterization using the trogocytosis-based assay^4^. These studies have established a standardized methodology that utilizes biotin-streptavidin staining to identify lymphocytes that perform trogocytosis.

In 2019, Li et al. reported a T-cell antigen discovery method that involved trogocytosis^93^. The authors engineered Jurkat T cells with F5-TCR or 1G4-TCR and K562 leukemia cells with their corresponding antigens, MART1 and NYESO1, via human leukocyte antigen A2 (HLA-A2). Single-chain trimer (SCT) technology enables the high expression of these antigens in K562 cells. Co-culturing these cells confirmed that trogocytosis is antigen-specific and depends on SCT affinity. The assay demonstrated sufficient sensitivity to isolate target cells even when diluted at a ratio of 1:10,000 with non-expressing cells. This method was extended to identify tumor neoantigens using an SCT library in K526 cells, co-cultured with Jurkat T cells. The neoepitopes were identified by next-generation sequencing and validated by T-cell cytotoxicity, which demonstrated the strategic significance of trogocytosis for neoantigen identification.

Similar research has been conducted to isolate patient-derived tumor antigen-specific TCRs^94^. Although previous studies focused on the tumor antigen itself, this study shifted its focus to TCRs for therapeutic purposes. Specifically, tumor-associated antigen (TAA)-specific T cells were isolated from 39 patients who received hematopoietic stem cell transplantation. The researchers isolated exhausted T-cell populations that express inhibitory receptors (IR), such as PD1 and Tim3. TCRs derived from the IR-positive population resulted in efficient lysis of target cells when incorporated into CD8 T cells. These data suggest that exhausted T cells exhibit TCRs highly specific to TAAs. By employing a trogocytosis-based method with ligandome-on-chip technology, the authors successfully identified patient-derived TAA-specific TCRs on CD8 T cells.

Wang et al. devised a synthetic intercellular delivery system inspired by trogocytosis^95^. They developed farnesylated chemically self-assembled nanorings (fCSANs), which can stably bind to the surface of sender cells prior to trogocytosis. Upon interaction between the sender and receiver cells, fCSANs were transferred to the receiver cell via trogocytosis. By loading a pro-apoptotic drug, monomethyl auristatin E, onto fCSAN, they observed successful delivery of the drug to the receiver followed by apoptosis. This approach suggests a novel cell-specific, intercellular drug delivery system that does not require genetic modification of either the sender or receiver cells.

Studies have developed trogocytosis-based methodologies for identifying cellular properties, including tumor neoantigens and their cognate TCRs, which are useful for discovering immunotherapy targets. Moreover, trogocytosis-inspired biomimetic technology represents a new method to selectively deliver immunotherapeutic drugs to target cells. These findings collectively highlight the potential of trogocytosis in advancing cancer biology and immunotherapy.

## Discussion

Trogocytosis, a process of membrane exchange between cells, plays a crucial role in immune regulation within the tumor microenvironment. Unlike phagocytosis, it preserves the function of transferred molecules and impacts the functionality of T cells, NK cells, macrophages, and monocytes. This process can either enhance or suppress immune responses in a context-dependent manner. T cells acquire antigen presentation capabilities from dendritic cells and macrophages. Additionally, T cells acquire immune-suppressive molecules, such as HLA-G and PD-L1, to create the tumor microenvironment. Trogocytosis also affects therapeutic strategies, particularly CAR-T-cell therapy, by causing antigen loss from target cancer cells, thereby reducing treatment efficacy. Future research should focus on understanding the mechanisms of trogocytosis and exploring how the manipulation of this process could enhance cancer immunotherapy. In summary, trogocytosis has a dual role in anti-tumor immunity, and further research could significantly improve cancer treatments.
